# A rare presentation of appendicitis as groin swelling: a case report

**DOI:** 10.1186/1757-1626-2-53

**Published:** 2009-01-14

**Authors:** Maheswaran Pitchaimuthu, Stephen Dace

**Affiliations:** 1 Department of General Surgery, Altnagelvin Area Hospital, Londonderry, Northern Ireland, UK; 2 SpR in HPB Surgery, Imperial College Healthcare NHS Trust, Hammersmith Hospital, London, W12 0HS, UK; 3 Consultant Surgeon, Western Health and Social Care Trust, Altnagelvin Hospital, Londonderry, BT47 6SB, UK

## Abstract

**Background:**

Appendicitis in femoral hernia is a rare condition, which raises diagnostic challenge.

**Case Report:**

A 40-year-old man presented with painful right-sided groin swelling of 1-week duration. The area was explored, with presumpted diagnosis of inguinal abscess. At exploration a femoral hernia was found which contained a mildly inflammed appendix. Appendicectomy and hernia repair was done. Post surgical course was uneventful. We present this case with brief summary of literature pertaining to such lesions.

**Discussion:**

The rare occurrence of femoral hernia containing appendix may be explained by different degrees of intestinal rotation during development or variation in its attachment to the caecum. Inflammation is due to tight femoral ring. Preoperative diagnosis is difficult. Management options are diverse.

**Conclusion:**

We present this case because of rarity. Early surgery prevents complications.

## Introduction

Inflammatory swellings of the groin are common, and the changes are often attributed to infection. Although this is possible, inflammatory swellings are often secondary to groin hernia. We present an unusual case of groin swelling, outlining its investigations and subsequent management.

## Case report

An otherwise healthy 40-year-old male presented with a one-week history of pain and swelling in his right groin. There was no history of trauma or previous hernia, and his bowel habit was normal. On examination he had a right inguinal swelling (6 × 3 cm) lateral to the pubic tubercle. There was no evidence of a cough impulse.

**Figure 1:**
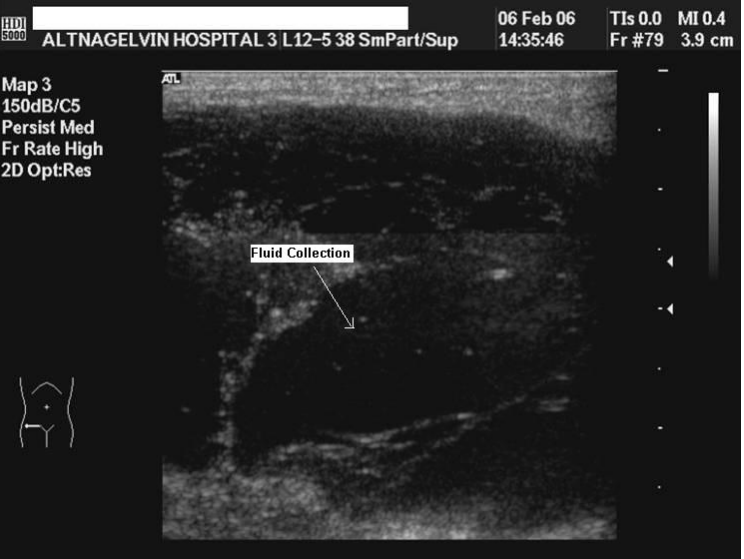
**USS right groin showing fluid collection**.

An ultrasound of the region was performed (Figure 1), which showed evidence of cellulitis and a fluid collection. The fluid was aspirated; it was blood stained with no evidence of pus. Routine blood tests were normal.

**Figure 2:**
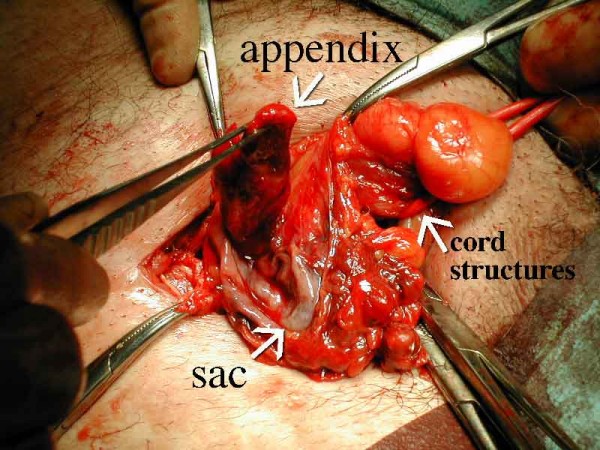
**Intra operative picture showing appendix in femoral hernia sac**.

Given the clinical findings, he was taken to the operating theatre for exploration. A standard oblique groin incision was used, and an incarcerated femoral hernia was identified. The back of the inguinal canal and the neck of the sac were opened. Inside the sac, a long, mildly inflamed appendix was found (Figure 2). An appendicectomy was performed and the excess sac excised and transfixed. Given that there was minimal inflammation of the appendix and there was no obvious evidence of infection outside the sac, the back wall of the inguinal canal was repaired using the Lichtenstein tension-free mesh method, using 15 × 7 cm Vypro Mesh and 2-0 prolene. The patient had an uneventful recovery.

## Discussion

The main pathologic conditions manifesting as masses in the groin fall into five major groups: congenital abnormalities, non-congenital hernias, vascular conditions, infectious or inflammatory processes, and neoplasms [1,2]. The hernial sac may contain preperitoneal fat, omentum, colon, or small bowel but reports of femoral hernia containing the vermiform appendix are rare, reported to occur in 0.8% of femoral hernia [3]. Rence Jacques Croissant de Garengeot, an 18^th ^century Paris Surgeon, was the first to describe appendix in femoral hernia [4]. Appendix in left femoral hernia [5], carcinoid tumour of appendix [6] and stomach [7] as femoral hernia contents have also been reported in the past. Factors contributing to this condition include degrees of intestinal malrotation or the presence of an abnormally large caecum which extends into the pelvis [3].

It is very difficult to diagnose the presence of an appendix within a femoral hernial sac, and to date, only one case has been diagnosed prior to surgery. This was identified at CT scan [8].

Tight femoral ring leads to strangulation and appendicitis. Appendicitis in a femoral hernia does not usually lead to abdominal peritonitis, due to narrow hernia sac neck which prevents inflammation of the parietal peritoneum. Clinical signs include local groin swelling, inflammation and spreading cellulitis, but often the patient feels generally well with no systemic features of sepsis, as in this case [3].

If left untreated, the inflammation may resolve or lead to complications including abscess [9], necrotizing fasciitis [10], necrosis of hernial contents [11] and development of bowel obstruction [12] and even death.

Due to the rarity of such cases treatment options remain diverse. Each case should be judged separately, and treatment based on the principles of removing the source of sepsis (either operatively or by aspiration) should be employed [13,14].

## Conclusion

We present a case of acute appendicitis complicating an incarcerated femoral hernia. As is often the case, the diagnosis was made at surgery. By following the principles of removing the source of sepsis and repairing the hernial defect, the patient made a safe recovery from a potentially serious condition.

We present this case because of appendix in femoral hernia that too in a young male patient is very rare. Early surgical treatment prevents potential complications.

## Consent

A written informed consent was obtained from the patient for publication of this case report and accompanying images. A copy of the written consent is available for review by the Editor in Chief of this journal.

## Competing interests

The authors declare that they have no competing interests.

## Authors' contributions

MP was the oncall registrar, who performed the surgery and a primary author for this manuscript. SD was the oncall consultant who was a contributor in writing the manuscript.

## References

[B1] Shadbolt CL Heinze SBJ Dietrich RB Imaging of Groin Masses: inguinal anatomy and pathologic conditions revisited RadioGraphics 2001 21 s261 71 11598262 10.1148/radiographics.21.suppl_1.g01oc17s261

[B2] Apostolidis S Papavramidis TS Michalopoulos A Papadopoulos VN Paramythiotis D Harlaftis N Groin Swelling, the Anatomic Way Out of Abdominal Haematomas: a Case Report and Explicative Literature Review Acta Chir Belg 2008 108 251 253 18557154 10.1080/00015458.2008.11680214

[B3] Nguyen ET Komenaka IK Strangulated femoral hernia containing a perforated appendix Can J Surg 2004 47 1 68 9 14997930 PMC3211803

[B4] Akopian G Alexander M De Garengeot hernia: appendicitis within a femoral hernia Am Surg 2005 71 6 526 7 16044937

[B5] Scepi M Richer JP Muller J Appendix in a left crural herniated position: apropos of a case. Explanation by human ontogenesis J Chir (Paris) 1993 130 11 479 82 8163605

[B6] Ivicic J Zaloudik J Carcinoid of the appendix in incarcerated femoral hernia Rozhl Chir 1999 78 7 359 61 10596574

[B7] Isaacs LE Felsenstein CH Acute appendicitis in a femoral hernia: an unusual presentation of a groin mass J Emerg Med 2002 23 1 15 8 10.1016/S0736-4679(02)00455-9 12217466

[B8] Lane MJ Liu DM Huynh MD Jeffrey RB Jr Mindelzum RE Kats DS Suspected acute appendicitis: non enhanced helical CT in 300 consecutive patients Radiology 1999 213 341 6 10551210 10.1148/radiology.213.2.r99nv44341

[B9] el Mansari O Sakit F Janati MI Acute appendicitis on crural hernia Presse Med 2002 31 24 1129 30 French 12162097

[B10] Guirguis EM Taylor GA Chadwick CD Femoral appendicitis: an unusual case Can J Surg 1989 32 5 380 1 2766144

[B11] Naude GP Ocon S Bongard F Femoral hernia: the dire consequences of a missed diagnosis Am J Emerg Med 1997 15 7 680 2 10.1016/S0735-6757(97)90184-4 9375551

[B12] Wyatt JP Varma JS Femoral hernia appendix causing small intestinal obstruction Postgrad Med J 1992 68 797 223 4 1589385 10.1136/pgmj.68.797.223 PMC2399242

[B13] Delamarre J Descombes P Grillot G Deschepper B Deramond H Hydorcele of pancreatic origin. X-ray computed tomographic study of an intrascrotal collection in an acute outbreak of chronic pancreatitis Radiol 1988 69 11 689 90 3236292

[B14] Michalopoulos A Papadopoulos V Apostolitis S Papavramidis T Paramythiotis D Berovalis P A rare case of pancreatic pseudo-cyst masquerading as hydrocele Acta Gastroenterol Belg 2006 69 424 17343088

